# FCVGAN: A Novel Generative Framework Integrating Fuzzy C-Means Clustering with Conditional VAE-GAN for Polyp Segmentation

**DOI:** 10.3390/s26196095

**Published:** 2026-09-25

**Authors:** Nabil Marzoug, Moulay A. Akhloufi

**Affiliations:** Perception, Robotics, and Intelligent Machines (PRIME), Department of Computer Science, Université de Moncton, Moncton, NB E1A 3E9, Canada; moulay.akhloufi@umoncton.ca

**Keywords:** polyp segmentation, data augmentation, generative adversarial networks, conditional VAE, fuzzy C-Means clustering, medical image synthesis, transfer learning, deep learning

## Abstract

Early detection of colorectal cancer is of paramount importance. Polyp segmentation using deep learning models can play an important role in this process; however, these models require large annotated datasets that are difficult to obtain in medical imaging. In this work, we propose FCVGAN (Fuzzy C-Means Conditional Variational Autoencoder Generative Adversarial Network), a mask-conditional generative framework that combines Fuzzy C-Means clustering, a Conditional VAE, and a SPADE-based GAN with a multi-scale discriminator. We evaluate two augmentation strategies across four polyp datasets: per-dataset training and transfer learning from Kvasir-SEG. The segmentation experiments repeated across five independent seeds show that transfer augmentation is the strategy that achieved the highest mean Dice score on three of the four datasets, while per-dataset augmentation performed best on Kvasir-Sessile. Compared with the corresponding baselines, transfer augmentation improved the mean Dice score by 2.4%, 0.8%, 16.0%, and 6.2% on Kvasir-SEG, CVC-ClinicDB, Kvasir-Sessile, and ETIS-Larib, respectively. The statistical analysis also showed large effect sizes for transfer augmentation over the baseline across all datasets. When performing the generation quality analysis, per-dataset training achieved a better average FID. However, the images generated with transfer learning showed lower similarity to the training data, which suggests that visual fidelity alone is not a complete indicator of augmentation effectiveness. The ablation study across the five configurations also showed that the contribution of the FCVGAN components depends on the dataset, with the full FCVGAN model providing the most balanced performance across the evaluated datasets. Overall, FCVGAN provides an effective framework for synthetic polyp augmentation, while transfer learning offers a practical strategy for supporting multiple target datasets using a single trained generator.

## 1. Introduction

Colorectal cancer (CRC) is considered to be a major health issue and remains one of the most commonly diagnosed malignancies and the second leading cause of cancer death worldwide, with around 1.9 million new cases and about 930,000 deaths each year [1]. The majority of CRCs follow a well-known adenoma–carcinoma sequence, where benign precancerous lesions gradually develop into malignant tumors over about 10–15 years [2]. This extended progression window presents a critical opportunity for early intervention: when CRC is detected at a localized stage, the five-year survival rate exceeds 90%, whereas diagnosis at a distant (metastatic) stage reduces five-year survival to approximately 14% [3,4].

Colonoscopy is still the gold standard for detecting and removing polyps. From a sensing perspective, the endoscope can be considered as a visual sensing system, where optical components and image sensors continuously capture images and videos of the gastrointestinal tract in real time. The quality of these acquired images can vary, depending on illumination, motion, blur, noise, specular reflections, and differences between endoscopic devices. These variations can directly affect the quality of the visual information provided to automated computer-aided systems. Studies also report adenoma miss rates of 22–28%, with flat and sessile polyps being particularly prone to being missed [5].

Deep learning has been considered as a promising solution to support clinical decision-making in colonoscopy by automatically analyzing the images acquired from these endoscopic sensing systems. Convolutional neural networks (CNNs) have demonstrated strong performance in polyp detection and segmentation, with recent methods achieving Dice scores approaching 90% on benchmark datasets [6,7]. Several recent works in biomedical sensing have also investigated deep learning for automatic analysis of endoscopic images, showing the growing role of intelligent image processing in endoscopic sensing systems [8,9]. However, these models can still fail to generalize to unseen datasets acquired from different sources. This is mainly because they are often trained and evaluated on data from single centers or relatively homogeneous populations, leading to performance degradation when applied across different clinical environments and imaging conditions [10].

Poor generalization is caused by the limited size and diversity of annotated colonoscopy datasets. In contrast to natural-image tasks that provide millions of labeled samples, medical imaging datasets typically include only hundreds to a few thousand annotated images [11]. The most widely used polyp segmentation benchmarks are Kvasir-SEG (1000 images) [12], CVC-ClinicDB (612 images) [13], and ETIS-Larib (196 images) [14]. Collectively, they provide fewer than 2000 annotated samples, which is only a small fraction of what deep learning models typically need. Additionally, getting more data is neither cheap nor simple: expert annotation is time-consuming and expensive, patient privacy must be carefully protected, and the polyps that matter most clinically, flat and serrated lesions with higher malignant potential, are precisely the ones that appear least often in available datasets [15]. Traditional data augmentation techniques, including geometric transformations (rotation, flipping, scaling) and photometric adjustments (brightness, contrast), have been widely used for data augmentation. While being effective to some extent, as they can create variations of the data, these techniques cannot generate new samples with novel pathological or anatomical information [16]. Consequently, traditional augmentation fails to address the core challenge: the absence of diverse polyp morphologies, textures, and clinical presentations in the original dataset.

Variational Autoencoders (VAEs), introduced by Kingma [17], provide a probabilistic approach to learn structured latent representations, which enables controllable generation and smooth interpolation between samples. By introducing probabilistic latent variables, VAEs offer control over the generation process; however, their reliance on reconstruction-based objectives often leads to blurrier outputs. Generative Adversarial Networks (GANs), on the other hand, are generative models that can produce sharp and realistic images through adversarial training; however, they lack an explicit latent structure, which makes controlled generation more challenging [18]. Hybrid architectures that combine VAEs with GANs have shown promise in balancing sample diversity with image quality [19]. In medical imaging, both approaches have been successfully applied to synthesize brain MRIs, chest X-rays, and histopathology slides, with studies demonstrating improved downstream task performance when synthetic data augments real training sets [20,21,22]. For colonoscopy specifically, several works have explored GAN-based polyp synthesis. Adjei et al. [23] modified Pix2Pix to generate synthetic polyp images. As a result, they achieved improvements in segmentation performance. More recently, conditional GANs with semantic guidance have shown good results in controlling polyp location, size, and shape [22,24]. However, many existing approaches still generate samples with limited diversity, as they rely on deterministic mask-to-image mappings, rather than an explicit modeling of the underlying data distribution via a probabilistic latent space.

**Overview and contributions.** For polyp synthesis, existing methods depend on deterministic mappings which, for a given segmentation mask, tend to produce nearly the same image each time, which inherently limits the generation of diverse samples and fails to capture the rich textural variability observed in real data. The key insight is simple: although polyp images appear highly heterogeneous, their appearances tend to group into a limited set of recurring texture patterns. These patterns can be learned without manual annotation and then used to guide the synthesis of more diverse and realistic images. To the best of our knowledge, this is the first approach that integrates fuzzy clustering into the latent space of a VAE-GAN architecture for polyp data augmentation. Our main contributions are as follows. *(1) Texture-aware conditional synthesis.* In this study we introduce FCVGAN, a generative approach that integrates Fuzzy C-Means clustering within a conditional VAE-GAN. FCVGAN is able to separate texture characteristics from spatial structure as it learns cluster assignments alongside image generation. This allows it to produce diverse polyp appearances while still following the shape defined by the input mask. *(2) Hybrid generative architecture.* Our architecture is hybrid as it integrates different components, including FCM clustering within a conditional VAE-GAN with SPADE normalization. This design helps to achieve two main things: first, it helps preserve fine-grained details that are considered challenging in polyp synthesis. Second, the probabilistic latent representation allows the model to introduce variations in the generated images while preserving the spatial structure defined by the input mask. *(3) Comprehensive cross-dataset evaluation.* We conducted experiments using four publicly available polyp datasets. We evaluate both per-dataset augmentation and a transfer setting in which the generative model trained on Kvasir-SEG is directly applied to target datasets without fine-tuning. This allows us to study whether a single trained generator can produce useful synthetic samples across different target distributions. The downstream segmentation experiments were further repeated across five independent seeds to evaluate the stability of the observed improvements. *(4) Systematic ablation analysis.* Through an ablation study, we evaluate five model configurations: the full FCVGAN, FCM+GAN without the CVAE branch, CVAE-GAN without FCM clustering, CVAE without adversarial training, and GAN-only without both CVAE and FCM. The results show that the contribution of the different components depends on the target dataset. In particular, removing the CVAE reduces the average segmentation improvement from +13.92% to +7.29% and causes a substantial performance degradation on ETIS-Larib. Overall, the full FCVGAN provides the most balanced performance across the evaluated datasets.

## 2. Related Works

Firstly, we review generative models in medical image synthesis and data augmentation, addressing the challenges of data scarcity [25,26]. We review three major generative paradigms: variational autoencoders, generative adversarial networks and diffusion models, then we dive into the use of these generative approaches for polyp image synthesis. Beyond generative modeling, fuzzy learning frameworks have also been used to explicitly represent gradual membership and similarity in data-driven models. For example, Cacciola et al. [27] proposed a prototypical fuzzy similarity-based classification framework in which class membership is defined according to the similarity between samples and representative prototypes. Although their application differs from medical image synthesis, their work provides a methodological example of how fuzzy membership can be explicitly formalized within a learning framework.

### 2.1. Variational Autoencoders

Variational Autoencoders (VAEs) [17] have been widely used for medical image synthesis, either alone or combined with other generative approaches. Rais et al. and Yeoh et al. [28,29] provide comprehensive reviews of VAEs in medical imaging, in which they highlight the use of VAEs and their ability to generate synthetic images that expand limited datasets and balance class distributions.

VAEs have been successfully applied across medical imaging modalities, including abdominal CT synthesis [30], cardiac MRI generation [31], and brain imaging [32]. Hybrid architectures combining VAEs and GANs have been widely used also in several works [19]. These architectures aim to incorporate the latent space of the VAE into the adversarial training of GAN. These approaches achieve both sample diversity and image quality due to the hybrid combination of both architectures [33,34]. Ref. [33] demonstrated that VAE-GAN hybrids achieve both sample diversity and image quality for medical image synthesis. For endoscopy specifically, Diamantis et al. [35] proposed EndoVAE, a generative approach that generates endoscopic images, showing that VAE-based augmentation can improve model performance while avoiding the training instability associated with GANs.

### 2.2. Generative Adversarial Networks

As generative adversarial networks (GANs) [18] have become a leading approach for generating high-fidelity images, several approaches have been used that exploit GANs for medical image synthesis. Frid-Adar et al. [20] demonstrated that GAN-based synthetic data augmentation significantly improves CNN performance in liver lesion classification, establishing a paradigm for medical image augmentation. Subsequent reviews have documented extensive GAN applications across radiology, pathology, and dermatology [22,36,37]. Conditional GANs are an extended version of GANs that incorporate auxiliary information for data synthesis. Pix2Pix [38] introduced paired image-to-image translation using U-Net generators and PatchGAN discriminators. Pix2PixHD [39] extended this to high-resolution synthesis through the use of multi-scale generators and feature-matching loss. SPADE [40] proposed spatially adaptive normalization for semantic image synthesis, a technique in which modulation parameters are derived from input masks at each layer, basically preventing the loss of semantic information that occurs with conventional normalization. Studies confirm that models using SPADE tend to produce images with high quality that are well-aligned with input segmentation masks [41]. For medical imaging with limited data, Zhang et al. [42] proposed MinimalGAN, an image-to-image network that decouples style from content during self-encoding to synthesize diverse medical images with minimal training data. The approach introduces two synthesis techniques: style transfer, which preserves structural content while varying appearance, and target variation, which applies geometric transformations selectively to annotated regions rather than entire images. On the DRIVE retinal fundus dataset (20 training images), MinimalGAN achieved a style transfer deception rate of 0.405, significantly outperforming traditional methods such as AdaIN (0.193) and GauGAN (0.050). For retinal vessel segmentation, training with synthetic data alone improved sensitivity by 1.73% and F1 score by 0.6%, while combining real and synthetic data improved sensitivity by 3.2%. For polyp segmentation on CVC-ClinicDB, augmentation with synthetic images improved Dice score from 0.922 to 0.926 on the test set and from 0.691 to 0.728 on the cross-dataset CVC-ColonDB evaluation, demonstrating effective generalization from limited training samples. Training stability techniques including label smoothing, feature matching, and differentiable augmentation have been adopted to improve GAN performance on small medical datasets [43]. Despite these advances, GANs still face some challenges, including the problem of training instability, mode collapse, and limited diversity when trained on small datasets [41].

### 2.3. Diffusion Models

Diffusion models are considered to be a modern and powerful generative approach. They were introduced by Jascha Sohl-Dickstein et al. in 2015 [44]. Compared to other models (VAEs, GANs) they have demonstrated considerable improvement [45]. These models learn to reverse a gradual noising process, generating high-quality samples through iterative denoising. Latent Diffusion Models (LDMs) [46] reduce computational cost by applying diffusion in a compressed latent space, which enables high-resolution synthesis with practical training requirements. For medical imaging, Pinaya et al. [47] used latent diffusion models (LDMs) conditioned on clinical variables to generate high-resolution 3D brain MRI scans. Konz et al. [48] proposed diffusion that is guided with segmentation for anatomically controllable medical image generation, showing improved faithfulness to input anatomical masks compared to SPADE-based methods. Diffusion models have also been applied to cross-modality synthesis and image reconstruction tasks [45]. However, diffusion models have higher computational costs than GANs during both training and inference, which requires hundreds of denoising steps for sample generation. Recent work on accelerated sampling [49] and consistency models [50] addresses this limitation, however the practical deployment for real-time applications remains challenging for this type of algorithm.

### 2.4. Polyp Image Synthesis

Colonoscopy image generation is considered to be a hard task due to the high variability in polyp appearance, lighting conditions, and mucosal backgrounds. From a sensing perspective, colonoscopy images are acquired through endoscopic visual sensing systems, where optical components and image sensors continuously capture the gastrointestinal tract. The quality and appearance of these images can therefore vary depending on illumination, motion, blur, noise, specular reflections, and differences between endoscopic devices. These variations represent an important challenge for automated polyp analysis, as deep learning models directly rely on the information captured by these sensing systems. Recent works in *Sensors* have also investigated deep learning for automatic polyp detection and segmentation from colonoscopy images, showing the increasing role of intelligent image processing in endoscopic sensing applications [8,9]. In this context, synthetic image generation can help increase the diversity of the available training data by reproducing different polyp appearances and imaging conditions, which can improve the robustness of downstream detection and segmentation models. Early approaches adapted conditional GANs for mask-to-polyp generation. Shin et al. [51] address the lack of labeled polyp images for training detection models in colonoscopy. The authors have proposed a conditional GAN-based framework to generate realistic synthetic polyp images, with the goal of using them as data augmentation for polyp detection. Their key idea is that using only a binary polyp mask is not enough to generate realistic colonoscopy frames, so they combine the mask with edge information extracted from colonoscopy images. This helps preserve background structure and makes the synthetic images more realistic. They then test whether these generated images improve downstream polyp detection using Faster R-CNN. Adjei et al. [23] studied whether synthetic colonoscopy images with polyps can improve downstream polyp segmentation and detection. Instead of simply inserting synthetic polyps into existing images, the authors generate entirely new colonoscopy images conditioned on segmentation masks. They then use these synthetic samples as augmentation data and evaluate their effect on U-Net for segmentation and Faster R-CNN for detection. The main conclusion is that GAN-based augmentation is useful, especially when combined with traditional augmentation.

Several studies have explored different ways of conditioning generative models for polyp image synthesis. Qadir et al. [24] used segmentation masks to control explicitly the different characteristics of the generated polyp like the location, the size, and the shape. Song et al. [52] introduced their approach, called SemanticPolypGAN, for semantic generation of polyp shape and texture without additional input preparation. Yoon et al. [53] also have proposed a GAN-based approach designed for sessile serrated lesions (SSLs). The results showed that their model can improve the detection of these polyps, which are known to be frequently missed. More recently, diffusion models have been applied to polyp synthesis. Du et al. [54] have proposed ArSDM, an adaptive refinement semantic diffusion model that uses segmentation mask conditioning with loss reweighting to improve polyp segmentation and polyp detection. Their method conditions the diffusion process on the ground truth segmentation masks, introduces an adaptive loss re-weighting strategy to address the imbalance between small polyp regions and large background areas, and then refines generation using a pretrained PraNet model to enforce better alignment between synthesized images and their masks. They used five public benchmarks for evaluation and the generated samples have improved both segmentation and detection performance. For segmentation, training with ArSDM-generated data increased the overall performance of PraNet from 74.0% to 80.0% in mDice and from 67.5% to 73.2% in mIoU, while SANet improved from 79.4% to 81.5% in mDice and from 71.4% to 74.1% in mIoU. Dorjsembe et al. [55] developed Polyp-DDPM that incorporates masks as channel-wise conditioning. Their method generates synthetic polyp images by conditioning the diffusion process directly on binary segmentation masks through channel-wise concatenation, which allows the model to produce diverse images that remain consistent with the target abnormal region. Using the Kvasir-SEG dataset, Polyp-DDPM outperformed both SinGAN-Seg and a latent diffusion model in image quality, achieving the best FID/KID scores of 78.47/0.0704 on Kvasir-SEG and 81.10/0.0755 on HyperKvasir. The synthetic images have resulted in improved segmentation results. For example, when UNet++ was trained only on 900 Polyp-DDPM-generated images, it achieved an IoU of 0.7156 and an F1 score of 0.8342 on the Kvasir-SEG test set, surpassing training on 900 real images (0.7067 IoU, 0.8281 F1). On the unseen HyperKvasir dataset, the same model reached 0.7739 IoU and 0.8725 F1, and on ETIS-LaribPolypDB it achieved 0.5295 IoU and 0.6923 F1, outperforming the other synthetic-data baselines across all three segmentation backbones. Ref. [56] introduced ControlPolypNet, a ControlNet-based framework for controlled polyp synthesis that uses custom masks to regulate the shape, size, and location of the generated lesions while preserving the surrounding endoluminal scene. To improve the clinical usefulness of the generated samples, the framework also incorporates a filtering step based on a pretrained detector to discard synthetic images that do not exhibit lesion-characterizing features. When used for data augmentation in downstream polyp segmentation, the selected synthetic images led to average improvements of 6.84% and 1.3% in Jaccard index on CVC-ClinicDB and Kvasir-SEG, respectively, showing that controlled and clinically screened synthetic data can effectively enhance segmentation performance. Beyond generative methods, and in a different approach that addresses polyp segmentation challenge through uncertainty-aware preprocessing, Hu et al. [57] have proposed NeutSS-PLP, which is a framework for colorectal polyp extraction that combines neutrosophic image enhancement with a saliency detection network. Their method uses neutrosophic enhancement to basically reduce bright specular reflections that can make polyp boundaries harder to detect. The enhanced images then are processed by a saliency detection network that combines features from different levels and scales to improve polyp localization and segmentation.

## 3. Methodology

We present FCVGAN, a hybrid architecture that integrates Fuzzy C-Means clustering into a conditional VAE-GAN framework for mask-conditional polyp image synthesis. Figure 1 illustrates the complete architecture comprising three main components: an encoder with integrated FCM clustering, a SPADE-based generator, and a multi-scale discriminator.

### 3.1. Problem Formulation

Given a binary segmentation mask $m\in {0,1}^{H\times W}$, our objective is to generate a realistic colonoscopy image $\hat{x}\in R^{3\times H\times W}$ that follows the spatial structure defined by the input polyp mask. Rather than learning a single deterministic mapping from mask to image, the model learns the conditional distribution p(x|m), which makes it possible to produce multiple realistic images from the same mask. The latent code of the proposed architecture has two main components: a variational component $z_{\mathrm{vae}}$ capturing continuous appearance variations, and a clustering component $z_{\mathrm{fcm}}$ encoding discrete texture patterns.

### 3.2. Encoder

The encoder *E* maps the image and the mask pair to a structured latent representation (Figure 1A). Then, the concatenated input $\left[x;m\right]\in R^{4\times 256\times 256}$ is processed through six convolutional blocks with channel dimensions 64→128→256→512→512→512, each using 4×4 kernels with stride 2, batch normalization, and LeakyReLU. The resulting features are flattened to $f\in R^{8192}$.

#### 3.2.1. Variational Latent Space

Two linear layers project f to the mean μ and log-variance $\log\sigma^{2}$ of a Gaussian posterior. The latent code is sampled via reparameterization:

(1) $$z_{\mathrm{vae}}=\mu +\sigma ⊙\epsilon ,\;\epsilon ∼N\left(0,I\right)$$

#### 3.2.2. Fuzzy C-Means Module

A third linear layer projects *f* to a clustering space $f_{\mathrm{fcm}}\in R^{128}$. We use K=12 clusters with a fuzzification parameter m=2. The cluster centers ${\left\{c_{k}\right\}}_{k=1}^{K}$ are initialized from a Gaussian distribution $N(0,0.01^{2})$ and are treated as learnable parameters.

For each feature representation $f_{\mathrm{fcm},i}$, we first compute the Euclidean distance $d_{ik}$ between the feature and each cluster center $c_{k}$. The fuzzy membership of sample *i* to cluster *k* is then computed as:

(2) $$u_{ik}=\frac{d_{ik}^{-\frac{2}{m-1}}}{\sum_{j=1}^{K}d_{ij}^{-\frac{2}{m-1}}},$$

where m>1 controls the degree of fuzziness and is fixed to m=2 in our experiments. In the implementation, this expression is computed as a softmax over $-\frac{2}{m-1}\log\left(d_{ik}\right)$ for numerical stability. Therefore, the softmax operation is only used to implement the FCM membership equation and does not introduce an additional temperature parameter τ.

The FCM latent representation is then obtained as the weighted combination of the cluster centers:

(3) $$z_{\mathrm{fcm},i}=\sum_{k=1}^{K}u_{ik}c_{k}.$$

To encourage compact fuzzy clusters, we use the following FCM compactness loss:

(4) $$L_{\mathrm{FCM}}=\frac{1}{NK}\sum_{i=1}^{N}\sum_{k=1}^{K}u_{ik}^{m}d_{ik}.$$

We additionally use an entropy regularization term to avoid the concentration of all samples into a limited number of clusters. Let ${\bar{u}}_{k}=\frac{1}{N}\sum_{i=1}^{N}u_{ik}$ denote the average membership of cluster *k*. The entropy regularization is defined as:

(5) $$L_{\mathrm{ent}}=\log K+\sum_{k=1}^{K}{\bar{u}}_{k}\log\left({\bar{u}}_{k}\right).$$

Unlike the conventional iterative FCM algorithm, the cluster centers in our architecture are not updated using alternating closed-form updates. Instead, they are optimized jointly with the encoder through backpropagation using the Adam optimizer. Therefore, the proposed module can be considered as a differentiable adaptation of FCM that allows fuzzy clustering to be integrated into the end-to-end generative framework.

### 3.3. Generator

The generator *G* synthesizes images from $z_{\mathrm{combined}}$ conditioned on mask *m* (Figure 1B). During training, $z_{\mathrm{combined}}$ is obtained from the encoder as described in Section 3.2. During transfer inference, the encoder is bypassed and $z_{\mathrm{combined}}$ is sampled directly from a standard Gaussian distribution, as detailed in Section 4.2. A fully connected layer projects the latent code to a 1024×4×4 feature map, which is progressively upsampled to 256×256 through seven SPADE residual blocks. Each SPADE block applies spatially adaptive normalization [40] where modulation parameters are derived from the input mask:

(6) $$\mathrm{SPADE}\left(h,m\right)=\gamma \left(m\right)⊙\frac{h-\mu (h)}{\sigma (h)}+\beta \left(m\right)$$

γ(m) and β(m) are produced by small convolutional networks taking the resized mask as input. This mechanism ensures polyp boundaries are preserved throughout generation. The final layer applies a 3×3 convolution with Tanh activation.

### 3.4. Multi-Scale Discriminator

We employ a multi-scale PatchGAN discriminator [39] operating at two scales (Figure 1C). Both discriminators receive the concatenation of image and mask as input. $D_{0}$ operates at full resolution (256×256), while $D_{1}$ operates at half resolution via average pooling. Each discriminator has convolutional layers with spectral normalization that produce patch-level predictions (30×30 and 14×14 respectively). This multi-scale evaluation provides complementary feedback on both fine textures and global structure.

### 3.5. Training Objectives

The training objective combines several loss terms to jointly optimize the generator, encoder, FCM module, and discriminator. We use the hinge loss for adversarial training. Since the discriminator operates at two different scales, the discriminator loss is computed as:

(7) $$L_{D}=\sum_{s=0}^{1}\left[E\left[\max(0,1-D_{s}\left(x,m\right))\right]+E\left[\max(0,1+D_{s}\left(\hat{x},m\right))\right]\right],$$

where *x* is the real image, $\hat{x}$ is the generated image, *m* is the segmentation mask, and $D_{s}$ represents the discriminator at scale *s*. The corresponding adversarial loss for the generator is defined as:

(8) $$L_{\mathrm{adv}}=-\sum_{s=0}^{1}E\left[D_{s}\left(\hat{x},m\right)\right].$$

We also use a feature matching loss to encourage the generated images to produce intermediate discriminator representations similar to those of real images. This loss is defined as:

(9) $$L_{\mathrm{FM}}=\frac{1}{S}\sum_{s=0}^{1}\sum_{l}{∥D_{s}^{\left(l\right)}\left(x,m\right)-D_{s}^{\left(l\right)}\left(\hat{x},m\right)∥}_{1},$$

where $D_{s}^{\left(l\right)}$ represents the feature representation from layer *l* of the discriminator at scale *s*, and S=2 is the number of discriminator scales.

To preserve the overall appearance of the original image, we include an $L_{1}$ reconstruction loss

(10) $$L_{\mathrm{recon}}={∥x-\hat{x}∥}_{1}.$$

We also use a VGG19-based perceptual loss to preserve higher-level visual features between the real and generated images:

(11) $$L_{\mathrm{perc}}=\sum_{l}w_{l}{∥\phi_{l}\left(x\right)-\phi_{l}\left(\hat{x}\right)∥}_{1},$$

where $\phi_{l}$ represents the features extracted from layer *l* of the pre-trained VGG19 network and $w_{l}$ represents the corresponding layer weight.

For the variational component, the KL divergence is used to regularize the latent distribution toward a standard Gaussian distribution:

(12) $$L_{\mathrm{KL}}=-\frac{1}{2}E\left[1+\log\sigma^{2}-\mu^{2}-\exp\left(\log\sigma^{2}\right)\right].$$

In addition, the FCM compactness loss $L_{\mathrm{FCM}}$ and the entropy regularization loss $L_{\mathrm{ent}}$, defined in Section 3.2.2, are included during training. The FCM loss encourages the latent features to remain close to their cluster centers according to their fuzzy memberships, while the entropy loss helps avoid the concentration of the samples into only a limited number of clusters.

The complete objective used to train the generator, encoder, and FCM module is therefore:

(13) $$\begin{array}{cc} L_{G}=\; & \lambda_{\mathrm{adv}}L_{\mathrm{adv}}+\lambda_{\mathrm{FM}}L_{\mathrm{FM}}+\lambda_{\mathrm{recon}}L_{\mathrm{recon}}\\ \; & +\lambda_{\mathrm{perc}}L_{\mathrm{perc}}+\lambda_{\mathrm{KL}}L_{\mathrm{KL}}+\lambda_{\mathrm{FCM}}L_{\mathrm{FCM}}+\lambda_{\mathrm{ent}}L_{\mathrm{ent}}. \end{array}$$

## 4. Experiments

### 4.1. Datasets

To evaluate the effectiveness of our proposed framework, we conducted experiments on four datasets for polyp segmentation. Figure 2 presents representative image–mask pairs from the four datasets:

- Kvasir-SEG [12] is a large-scale polyp segmentation dataset containing 1000 colono scopy images with corresponding pixel-wise ground truth masks. This dataset has considerable variation in polyp appearance, including differences in size, shape, color, and texture. The images have variable resolutions ranging from 332× 487 to 1920 × 1072 pixels. In our experiments, the Kvasir-SEG dataset is the source dataset for training the generative model.
- CVC-ClinicDB [13] contains 612 frames extracted from 29 colonoscopy video sequences. All images have a resolution of 384 × 288 pixels and were acquired at the Hospital Clinic of Barcelona. This dataset is widely used as a benchmark for polyp segmentation algorithms and contains polyps with varying sizes and morphological characteristics.
- Kvasir-Sessile [12] is a specialized dataset containing 196 images of sessile (flat) polyps. These polyps are particularly challenging to detect and segment due to their subtle elevation and similarity in appearance to the surrounding mucosa. Sessile polyps are clinically significant as they have a higher risk of malignancy compared to pedunculated polyps. This dataset provides an important test case for evaluating segmentation methods on difficult polyp morphologies.
- ETIS-Larib [14] consists of 196 high-resolution colonoscopy images (1225 × 966 pixels). This dataset is considered one of the most challenging benchmarks due to significant variations in polyp size, shape, texture, and the presence of artifacts such as specular reflections and motion blur.

Table 1 summarizes the key characteristics of each dataset. For all experiments, we applied stratified sampling based on polyp size categories to ensure balanced representation in the training, validation, and test splits. Polyp size was computed as the ratio of polyp pixels to total image pixels, with samples that were grouped into small (<5%), medium (5–15%), or large (>15%). Each dataset was then divided into training (70%), validation (15%), and test (15%) sets, while preserving the same polyp-size distribution across splits to avoid the bias toward specific morphologies.

### 4.2. Implementation Details

All experiments were implemented in PyTorch 2.0 and conducted on a single NVIDIA GPU with CUDA acceleration. Mixed-precision training using automatic mixed precision (AMP) was employed to reduce the memory consumption and accelerate computation without compromising model performance.

The generator was trained using the Adam optimizer with a learning rate $1\times 10^{-4}$, while the discriminator used learning rate $4\times 10^{-4}$, both with $\beta_{1}=0.0$ and $\beta_{2}=0.999$. Loss weights were set as $\lambda_{adv}=1.0$, $\lambda_{FM}=10.0$, $\lambda_{recon}=10.0$, $\lambda_{perc}=10.0$, $\lambda_{KL}=0.05$, $\lambda_{FCM}=0.5$, and $\lambda_{ent}=0.01$. The number of epochs was set to 150 epochs with early stopping based on validation reconstruction loss, using a patience of 20 epochs. The batch size was set to 16.

For the downstream segmentation task, we employed UNet++ with an encoder featuring five levels of 32, 64, 128, 256, and 512 channels respectively. The segmentation model was trained using the Adam optimizer with learning rate $1\times 10^{-4}$ and a combined loss function of Dice loss and binary cross-entropy with equal weights. Also, for the segmentation, the number of epochs was set to 150 epochs with early stopping based on validation Dice score (patience of 20 epochs). A learning rate scheduler reduced the learning rate by a factor 0.5 when validation performance plateaued for 5 consecutive epochs.

All images were resized to 256 × 256 pixels and normalized to the range [−1,1] for generative model training. For segmentation, images were normalized using ImageNet statistics (mean = [0.485, 0.456, 0.406], std = [0.229, 0.224, 0.225]). For data augmentation during training, we included random horizontal flipping, random vertical flipping, and random rotation by multiples of 90 degrees, applied consistently to both images and masks.

For synthetic data generation, we generated one synthetic image per training mask, so we doubled the training set size. In the transfer learning protocol, the generative model trained on Kvasir-SEG was directly applied to the target datasets without any fine-tuning. In this setting, the target image is not required and the encoder is bypassed. Instead, a 256-dimensional latent vector is sampled directly from a standard Gaussian distribution, $z_{\mathrm{combined}}∼N\left(0,I\right)$, and then provided to the generator together with the target segmentation mask, such that $\hat{x}=G\left(z_{\mathrm{combined}},m\right)$. Therefore, the segmentation mask is the only input required from the target dataset, while the latent vector is sampled internally. The mask controls the spatial structure of the generated image through the SPADE layers, while the sampled latent vector introduces appearance variation. In this transfer setting, the encoder and the FCM module are not explicitly used during inference.

### 4.3. Computational Cost and Efficiency

We performed an analysis of the computational requirements to assess the practical efficiency and deployability of our proposed architecture FCVGAN. Table 2 presents the parameter distribution across the main architectural components. The encoder *E* has 14.25 M parameters: 11.1 M for convolutional feature extraction, 1.05 M for the FCM module (clustering layer and projection), and 2.1 M for the VAE latent projections (μ and $\log\sigma^{2}$ heads). The generator *G* (SPADE decoder) accounts for 96.2 M parameters and represents the largest component of the architecture due to the repeated high-dimensional convolutional operations and learnable modulation networks required for spatially adaptive normalization. The multi-scale discriminator *D* has 14.0 M parameters. Hence the total of parameters is 124.45 M trainable parameters during adversarial training.

The FCM module has only 1.05 M parameters, corresponding to approximately 0.84% of the total model. In contrast, the SPADE-based generator accounts for approximately 77% of the total parameter count, reflecting the computational capacity required for mask-conditioned image synthesis.

Table 3 shows the computational cost of each FCVGAN component in terms of floating-point operations (FLOPs) per forward pass. As presented in the table, the SPADE-based generator requires most of the computation, with 130.18 GFLOPs per forward pass, and the encoder requires 2.02 GFLOPs, while the discriminator requires 3.25 GFLOPs. During transfer inference and as only the generator is used, the cost is 130.18 GFLOPs per generated image. In the per-dataset setting, the encoder is also used, which increases the cost slightly to 132.20 GFLOPs per image.

The training was performed on a single NVIDIA RTX 4090 GPU. On the Kvasir-SEG dataset, each epoch took about 20 s, and training stopped after 104 epochs using early stopping, for a total training time of about 35 min. Compared to other diffusion-based methods such as Polyp-DDPM and ArSDM, FCVGAN is more efficient during image generation because it produces a synthetic image in a single forward pass instead of using several denoising steps.

At inference, the required components depend on which generation strategy is used. In the per-dataset setting, the encoder is used to obtain $z_{\mathrm{FCM}}$ and $z_{\mathrm{VAE}}$, so both the encoder and generator are active, corresponding to 110.45M parameters. In the transfer setting, only the target segmentation mask is available. In this case, $z_{\mathrm{combined}}∼N\left(0,I\right)$ is sampled directly and passed to the generator together with the mask. The encoder is not used, reducing the active parameter count to 96.2M. In this setting, the FCM module is used during training to organize the latent representation and guide the learning of the generator, but it is not used directly during inference. In the transfer setting, FCVGAN generates one 256×256 synthetic image in about 18 ms, which corresponds to roughly 55 images per second. This makes it suitable for generating large numbers of synthetic samples efficiently. In contrast, diffusion-based models require several denoising steps for each generated image.

Although FCVGAN generates images quickly, the model remains relatively large, mainly because of the SPADE decoder. This may make it harder to use on devices with limited memory or computing resources. Future work could explore techniques such as knowledge distillation, channel pruning, or lighter SPADE designs to reduce the model size while maintaining image quality.

### 4.4. Evaluation Metrics

We evaluate our approach using metrics for both generation quality and downstream segmentation performance.

#### 4.4.1. Segmentation Metrics

For evaluation of segmentation performance we used two complementary metrics that capture different aspects of spatial overlap between predicted and ground truth masks. The Dice Similarity Coefficient (DSC), also known as the F1 score, measures the harmonic mean of precision and recall and is defined as:

(14) $$\mathrm{DSC}=\frac{2|P\cap G|}{\left|P|+|G\right|}$$

where *P* denotes the set of pixels predicted as polyp and *G* denotes the ground truth polyp pixels. The Dice coefficient ranges from 0 (no overlap) to 1 (perfect agreement) and is widely adopted in medical image segmentation due to its robustness to class imbalance.

We used also the Intersection over Union (IoU), also known as the Jaccard index, and it provides a stricter measure of overlap by computing the ratio of intersection to union:

(15) $$\mathrm{IoU}=\frac{\left|P\cap G\right|}{\left|P\cup G\right|}$$

The IoU metric is informative in particular for segmentation quality in clinical applications as it penalizes both false positives and false negatives more heavily than Dice.

#### 4.4.2. Generation Quality Metrics

To assess the quality of synthetic images, we use the following metrics:

(a) Fréchet Inception Distance (FID): measures the distributional similarity between real and generated images in the feature space of a pre-trained InceptionV3 network [58]. Lower FID indicates higher visual quality and realism:
  (16) $$\mathrm{FID}=\left|\right|\mu_{r}-\mu_{g}{\left|\right|}^{2}+\mathrm{Tr}\left(\Sigma_{r}+\Sigma_{g}-2{\left(\Sigma_{r}\Sigma_{g}\right)}^{1/2}\right)$$
  where $\left(\mu_{r},\Sigma_{r}\right)$ and $\left(\mu_{g},\Sigma_{g}\right)$ are the mean and covariance of real and generated feature distributions, respectively.
(b) Structural Similarity Index (SSIM): [59] measures perceptual similarity between image pairs based on luminance, contrast, and structure, ranging from −1 to 1, with higher values indicating greater similarity.
(c) Peak Signal-to-Noise Ratio (PSNR): quantifies reconstruction quality in decibels (dB), with higher values indicating lower pixel-wise distortion.

#### 4.4.3. Memorization Metric

Following [60], we evaluate potential memorization by computing the maximum Pearson correlation between each synthetic image and all real training images. For descriptive analysis, correlations above 0.9 are treated as a warning of strong similarity and potential memorization, while values below 0.8 indicate lower similarity to the training data. Pearson correlation is used here as a practical similarity indicator rather than as a complete test of memorization.

#### 4.4.4. Statistical Analysis

To evaluate the consistency of the segmentation improvements, all segmentation experiments were repeated using five independent random seeds. For each seed, we use the mean test Dice score as one independent observation, resulting in five observations for each experimental setting. The same seeds were used across the baseline, transfer augmentation, and per-dataset augmentation settings to enable paired comparisons.

Since the number of the independent runs is limited, we used the two-sided paired Wilcoxon signed-rank test instead of assuming a normal distribution. In addition to the *p*-values, we report the rank-biserial correlation as an effect size and 95% bootstrap confidence intervals for the Dice differences between the compared methods. A Bonferroni correction is also applied to account for multiple pairwise comparisons across the four datasets.

## 5. Results

We evaluate FCVGAN using two training strategies: (1) transfer learning, where the model is trained on Kvasir-SEG and applied to generate synthetic images for all datasets, and (2) per-dataset training, where separate models are trained for each dataset. Our evaluation includes qualitative visual assessment, downstream segmentation performance, generation quality metrics, memorization analysis, and also an ablation study, to provide a thorough characterization of the proposed approach.

Figure 3 presents synthetic polyp images generated by both approaches across all four datasets. As visually observed in the figure, there are distinct characteristics between the two strategies. The per-dataset approach (right panel) produces images that are similar to real ones with more accurate color reproduction and texture consistency. In contrast, in the transfer learning approach (left panel) we see noticeable color shifts and artifacts (yellowish or greenish), especially on target datasets such as CVC-ClinicDB and Kvasir-Sessile.

Despite these visual differences, both approaches have successfully preserved the structural characteristics that are defined by the input masks, generating anatomically plausible polyp images with appropriate boundaries and shapes. This observation gives an important question: does higher visual fidelity translate to better downstream task performance? The following subsections will try to give an answer based on segmentation experiments, generation quality metrics, and diversity analysis.

### 5.1. Segmentation Performance

We evaluate the impact of synthetic data augmentation on polyp segmentation using UNet++ as the backbone network. For each dataset, we compare baseline training (using only real images) against augmented training (combining real images with synthetic images generated by FCVGAN) with two augmentation strategies: transfer and per-dataset. All configurations were evaluated using identical train, validation, and test splits. The experiments were conducted across five independent random seeds and the results are reported as the mean Dice coefficient ± standard deviation across the five runs. Table 4 summarizes the segmentation performance obtained with the three training configurations across the four datasets.

Looking at the results presented on the table, we notice that both augmentation strategies have improved the mean Dice coefficient over the baseline across all of the four benchmark datasets with variation on the magnitude between datasets.

On the larger datasets, which are Kvasir-SEG and CVC-ClinicDB, the transfer strategy has achieved the highest segmentation performance. For Kvasir-SEG, transfer augmentation increased the mean Dice coefficient from 0.8318 to 0.8518, which corresponds to an improvement of 2.4%, compared with 1.7% for the per-dataset strategy. A similar trend was observed on CVC-ClinicDB, where transfer augmentation reached a Dice coefficient of 0.8887 compared with 0.8830 for per-dataset augmentation and 0.8812 for the baseline, and despite the fact that these improvements on these two datasets were relatively modest, both augmentation strategies maintained performance above the corresponding baseline models.

Larger improvements were observed on the smaller and more challenging Kvasir-Sessile and ETIS-Larib datasets. On the Kvasir-Sessile dataset, the per-dataset augmentation has achieved the largest improvement, increasing the mean Dice coefficient from 0.4015 to 0.4832, corresponding to a gain of 20.3%, and for the transfer augmentation, it also produced a substantial improvement on this dataset, reaching a Dice coefficient of 0.4659, or 16.0% above the baseline. On ETIS-Larib, transfer augmentation achieved the highest Dice coefficient of 0.7443, compared with 0.7388 for per-dataset augmentation and 0.7007 for the baseline, corresponding to improvements of 6.2% and 5.4%, respectively. These results show basically that synthetic augmentation provides its largest relative benefit when the baseline segmentation performance is lower and also the amount of available training data is limited.

The variability across the five independent training runs also differed between datasets. On the Kvasir-SEG and ETIS-Larib datasets, the augmented models showed lower standard deviations compared with the baseline, especially for the transfer strategy. However, this behavior was not observed consistently on CVC-ClinicDB and Kvasir-Sessile, where the augmented models have exhibited similar or greater variability. Therefore, the main benefit of FCVGAN augmentation is actually reflected in the increase in mean segmentation performance rather than in a uniform reduction in training variability.

When the two augmentation strategies are compared directly, their average relative improvements across the four datasets are similar, reaching approximately 6.4% for transfer augmentation and 6.9% for per-dataset augmentation. However, these averages hide an important difference in their behavior. The transfer augmentation has achieved the highest mean Dice coefficient on three of the four datasets, which are Kvasir-SEG, CVC-ClinicDB, and ETIS-Larib, whereas the per-dataset augmentation has achieved the best result in only one dataset which is the Kvasir-Sessile dataset. For that, and from this observation we can say the results support the use of transfer augmentation as the preferred strategy when the same generative framework is intended to support several target datasets as it achieved the best segmentation performance on three out of four datasets while avoiding the additional cost of training a separate generator for each domain. The per-dataset training can also be considered as a useful alternative when the target data present distinct characteristics and the additional training cost is acceptable.

### 5.2. Statistical Comparison of Augmentation Strategies

A statistical analysis was also performed across the five independent training runs to further examine the differences between the baseline, transfer, and per-dataset configurations, as summarized in Table 5. For each random seed, we obtained a single mean Dice coefficient on the fixed test set, and the comparisons were therefore performed at the training-run level, with results obtained using the same random seed treated as paired observations. As the number of runs is limited, a two-sided Wilcoxon signed-rank test was used without assuming normality of the paired differences. Three comparisons were considered for each dataset: baseline versus transfer, baseline versus per-dataset, and transfer versus per-dataset. The Wilcoxon statistic (*W*) and the corresponding raw *p*-values were reported, and a Bonferroni correction was applied across the 12 pairwise comparisons. To complement the hypothesis tests, the matched-pairs rank-biserial correlation ($r_{rb}$) was used to quantify the magnitude and direction of the paired effect. The mean paired Dice difference (ΔDice) and its 95% bootstrap confidence interval were also calculated. Finally, the confidence intervals were obtained by resampling the five seed-level paired differences and therefore they describe uncertainty across independent training runs rather than patient-level uncertainty.

None of the pairwise comparisons has reached statistical significance after correction for multiple comparisons. However, this result should be interpreted in relation to the limited number of independent training runs. With five observations, the smallest possible two-sided Wilcoxon *p*-value is 0.0625, which means that even if all five runs favor the same strategy, the test cannot reach p<0.05.

This was the case for Kvasir-SEG. Transfer augmentation improved the mean Dice coefficient by 0.0200 over the baseline, and all five paired runs favored the transfer strategy. This produced the maximum rank-biserial effect size of $r_{rb}=1.000$, while the Wilcoxon test gave p=0.0625. The confidence interval was also fully positive, [0.0105, 0.0285], supporting the consistency of the improvement.

A similar trend was observed on the other datasets. Transfer augmentation showed large positive effects over the baseline on CVC-ClinicDB ($r_{rb}=0.733$), Kvasir-Sessile ($r_{rb}=0.867$), and ETIS-Larib ($r_{rb}=0.733$). The largest mean improvements were observed on Kvasir-Sessile (ΔDice = 0.0644) and ETIS-Larib (ΔDice = 0.0437).

The per-dataset strategy also showed large positive effects over the baseline on the Kvasir-SEG, Kvasir-Sessile, and ETIS-Larib datasets. On the CVC-ClinicDB dataset, however, we see that the effect was negligible ($r_{rb}=-0.067$), with a very small mean difference of 0.0018 and a confidence interval that included zero.

The direct comparison between transfer and per-dataset augmentation showed smaller differences. Transfer was moderately favored on Kvasir-SEG and CVC-ClinicDB ($r_{rb}=-0.467$ for both), while per-dataset augmentation was moderately favored on Kvasir-Sessile ($r_{rb}=0.333$), and for the ETIS-Larib dataset, the effect was negligible ($r_{rb}=0.067$), which indicates no clear advantage for either strategy across the five runs.

Overall, the statistical results agree with the segmentation results reported in Table 4. Transfer augmentation showed a large positive effect over the baseline on all four datasets and achieved the highest mean Dice score on three of them.

Although no direct comparison between the transfer and per-dataset augmentation has reached statistical significance, the overall results support that the transfer augmentation is to be considered as the preferred practical strategy when one generator is intended to support multiple target datasets. Per-dataset training remains a useful alternative when a target dataset benefits from a dedicated generator, as observed on Kvasir-Sessile.

### 5.3. Robustness Under Degraded Image Conditions

To further evaluate our approach and to examine the behavior of the segmentation models under changes in image quality, we conducted a robustness experiment on the ETIS-Larib dataset. The baseline, transfer-augmented, and per-dataset-augmented models obtained from the five independent training runs were evaluated without any additional training. The ETIS-Larib was the dataset selected as it is considered as a challenging evaluation case and because of its relatively small training set and lower baseline segmentation performance.

We considered for this experiment, four types of image degradation: Gaussian blur, Gaussian noise, reduced brightness, and reduced contrast. Gaussian blur was applied with a radius of 1.5 and Gaussian noise with a standard deviation of 0.03. Brightness and contrast were reduced using factors of 0.75 and 0.70, respectively. These transformations were applied only to the test images, while the corresponding ground-truth masks remained unchanged. Figure 4 illustrates the different image conditions used in this experiment.

Robustness was evaluated using the Dice coefficient under both clean and degraded conditions. In addition to the absolute Dice score, the performance drop was calculated as

(17) $$\Delta_{\mathrm{drop}}=Dice_{\mathrm{clean}}-Dice_{\mathrm{degraded}},$$

where a smaller value indicates better preservation of the segmentation performance after degradation. Results are reported as the mean ± standard deviation across the five independent training runs and are summarized in Table 6.

As expected, all degradation conditions reduced segmentation performance compared with the clean images. However, the effect of the degradations varied across the three training configurations. Under the Gaussian blur degradation, the transfer strategy has achieved the highest Dice coefficient of 0.4957, compared with 0.4845 for the per-dataset strategy and 0.4206 for the baseline. Transfer also showed the smallest performance drop under this condition ($\Delta_{\mathrm{drop}}=0.2486$), which indicates better preservation of its clean performance.

Under Gaussian noise, the baseline model achieved the highest Dice coefficient (0.2113), followed by transfer (0.1899) and per-dataset augmentation (0.1739). Therefore, neither augmentation strategy improved robustness over the baseline under this type of degradation. However, when the two augmented models are compared directly, transfer augmentation remained more robust than the per-dataset strategy.

A different behavior was observed when the brightness and the contrast of the images were reduced. Under reduced brightness, the per-dataset model achieved the highest Dice coefficient (0.2482), followed by transfer (0.2008), while the baseline dropped to 0.0947. A similar pattern was observed under reduced contrast, where per-dataset augmentation reached 0.2851, transfer reached 0.2578, and the baseline decreased to 0.0567. The smaller performance drops obtained by the augmented models under these two conditions indicate a robustness benefit against intensity-related image degradation.

The comparison also between the two augmentation strategies also shows different robustness characteristics. Transfer augmentation achieved higher Dice coefficients than the per-dataset strategy under the clean, blurred, and noisy conditions. In contrast, the per-dataset strategy performed better under reduced brightness and reduced contrast, and this suggests that transfer augmentation provides a stronger general performance across several conditions, while the per-dataset training can offer an additional advantage under changes related to illumination and contrast.

Overall, the robustness experiment shows that the benefit of FCVGAN augmentation depends on the type of image degradation. Transfer augmentation provided the best overall performance on clean and blurred images and outperformed the per-dataset strategy under three of the five evaluated image conditions. The per-dataset augmentation was more effective under reduced brightness and the contrast degradations, while the Gaussian noise degradation remained the most challenging case for both of the augmented models. These results show that the proposed augmentation framework is robust under several image-quality variations, while also showing that no single augmentation strategy is uniformly superior under all types of degradation.

### 5.4. Generation Quality

To understand the factors contributing to segmentation improvements, we analyze the visual quality of generated images using three metrics: Fréchet Inception Distance (FID), Structural Similarity Index (SSIM), and Peak Signal-to-Noise Ratio (PSNR). Table 7 presents these metrics for both training strategies across all datasets.

Per-dataset training achieves substantially lower FID scores across most datasets, with an average of 135.48 compared to 173.43 for transfer learning. Lower FID indicates that the distribution of the generated images more closely matches the distribution of real images, reflecting higher visual fidelity. The difference is particularly noticeable on CVC-ClinicDB (107.27 vs. 189.37, a difference of 82.10) and Kvasir-SEG (68.91 vs. 126.58, a difference of 57.67). These results quantitatively support the visual observations presented in Figure 3, where per-dataset training produces images with more realistic colors and textures. The FID scores also reveal dataset-specific differences. Kvasir-SEG achieves the lowest FID score (68.91 for per-dataset training), indicating a closer distribution between the generated and real images. In contrast, ETIS-Larib shows the highest FID scores for both approaches (195.11 and 191.02), indicating greater difficulty in reproducing its image characteristics. Interestingly, transfer learning slightly outperforms per-dataset training on ETIS-Larib (191.02 vs. 195.11). This suggests that knowledge transferred from the larger Kvasir-SEG dataset can provide some benefit for generation on this challenging target dataset.

For SSIM and PSNR, moderate values were obtained for both approaches, ranging from 0.29 to 0.54 for SSIM and from 11.25 to 14.49 dB for PSNR. These metrics measure structural and pixel-level similarity between generated and real images. However, these values should be interpreted in the context of FCVGAN, which performs mask-conditional generation rather than reconstruction of a specific real image. Therefore, high pixel-wise similarity to an individual reference image is not necessarily expected. These metrics should consequently be considered together with FID, qualitative evaluation, and downstream segmentation performance when assessing generation quality.

A critical observation is obtained when comparing the generation quality with the segmentation performance. Despite achieving lower FID scores, the per-dataset training does not consistently lead to better downstream segmentation. Transfer augmentation achieved the highest mean Dice coefficient on three of the four datasets, while per-dataset training achieved the highest result on Kvasir-Sessile. This finding suggests that visual quality, as measured by FID, does not directly predict augmentation effectiveness. Other characteristics of the generated images, including their similarity to the original training samples and their diversity, may also influence their usefulness for segmentation augmentation. We investigate this relationship further through the memorization analysis presented in the following subsection.

### 5.5. Memorization Analysis

The observation that better visual quality does not necessarily translate into better segmentation performance motivates a deeper investigation of the diversity of the generated samples. Generative models, particularly when trained on limited datasets, may produce samples that closely resemble their training examples rather than introducing sufficiently new variations. In a data augmentation setting, such behavior can reduce the additional information provided to the downstream segmentation model.

Following the correlation-based analysis used by Akbar et al. [60], we quantified similarity by computing the maximum Pearson correlation between each synthetic image and all real training images. For each generated image, the highest correlation with any training image was retained. Therefore, lower values indicate that the generated image is less similar to its nearest training example, while high values indicate stronger similarity and a greater possibility of memorization. For descriptive interpretation, correlations below 0.8 were considered to indicate high diversity, values between 0.8 and 0.9 moderate similarity, and values above 0.9 were considered a warning of strong similarity to the training data.

Table 8 and Figure 5 present the results for the two training strategies. A clear difference can be observed between transfer and per-dataset training.

Transfer learning achieves lower mean maximum correlations across all four datasets, with an average of 0.767 compared with 0.926 for per-dataset training. The difference is particularly large on CVC-ClinicDB, where the mean correlation decreases from 0.948 for per-dataset training to 0.683 for transfer learning. Similar differences are observed on ETIS-Larib (0.931 vs. 0.726) and Kvasir-Sessile (0.899 vs. 0.771). On Kvasir-SEG, the difference is smaller, with correlations of 0.923 and 0.886 for per-dataset and transfer training, respectively.

The percentage of generated samples with a maximum correlation above 0.9 further illustrates this difference. For per-dataset training, 84.4% of Kvasir-SEG samples, 99.1% of CVC-ClinicDB samples, 49.3% of Kvasir-Sessile samples, and 86.7% of ETIS-Larib samples exceed this threshold. In contrast, the corresponding value for transfer learning is 35.9% on Kvasir-SEG and 0% on the three target datasets. These results indicate that the per-dataset models generally generate samples that are more similar to their training images, while transfer learning introduces greater image-level variation.

This observation provides an additional explanation for the relationship between generation quality and downstream segmentation. Although per-dataset training achieves better FID scores, its generated images also show stronger similarity to the corresponding training data. Transfer learning, in contrast, produces more diverse samples and achieved the highest mean segmentation Dice score on three of the four datasets. This suggests that visual fidelity alone is not sufficient to determine the usefulness of synthetic images for data augmentation. Diversity with respect to the original training data can also play an important role by introducing additional variations during segmentation training.

This behavior is particularly relevant for the smaller target datasets, where training a generative model using only the available target images provides a more limited amount of information from which to learn the image distribution. Transfer learning can reduce this limitation by first learning broader generative characteristics from the larger Kvasir-SEG dataset and then applying this learned representation to the target datasets.

It should be noted that the present analysis uses maximum pixel-level Pearson correlation as a practical indicator of similarity rather than as a complete test of memorization. Pearson correlation does not fully capture perceptual or feature-level similarity and does not separately analyze the polyp and background regions. A more extensive privacy or memorization assessment could include perceptual distances, deep feature-space similarity, nearest-neighbor visualization, and region-specific analysis. However, such analyses are beyond the scope of the present work. Here, the correlation analysis is used to provide a consistent comparison of the relative diversity produced by the two training strategies.

Finally, since FCVGAN performs mask-conditional generation, some structural similarity is naturally expected because the input mask constrains the spatial location and shape of the generated polyp. The relevant difference is therefore not the complete absence of similarity, but whether the model can preserve the required spatial structure while introducing variations in appearance, including texture, color, and local image details.

### 5.6. Ablation Study

To validate the contribution of each component in FCVGAN, we conduct an ablation experiment by systematically removing different architectural components. We have evaluated five configurations: (1) the full FCVGAN model, (2) FCM+GAN without the CVAE branch, (3) CVAE-GAN without FCM clustering, (4) CVAE without the GAN discriminator, and (5) GAN only without both CVAE and FCM. Table 9 presents the segmentation improvement obtained by the different configurations using the transfer learning approach.

To keep the computational cost of the ablation study manageable, each ablation configuration was evaluated using a single training run. Therefore, the results reported in Table 9 should be interpreted as a component-wise comparison under the same experimental setting rather than as the five-run averages reported in the main segmentation experiments.

The full FCVGAN model achieves an average improvement of +13.92% across the four datasets. Positive improvements are obtained on all datasets, including +2.98% on Kvasir-SEG, +1.24% on CVC-ClinicDB, +37.43% on Kvasir-Sessile, and +14.02% on ETIS-Larib. In particular, the improvement obtained on ETIS-Larib shows that the complete architecture can provide useful augmentation on this challenging target dataset.

To better isolate the contribution of the CVAE beyond the FCM component, we also evaluate an FCM+GAN configuration in which the CVAE branch is removed while keeping the FCM and adversarial components. This configuration achieves improvements of +0.67% on Kvasir-SEG, +1.75% on CVC-ClinicDB, and +45.74% on Kvasir-Sessile. However, its performance decreases considerably on ETIS-Larib, with a degradation of −19.01%. The average improvement is +7.29%, compared with +13.92% for the full FCVGAN.

A direct comparison between FCM+GAN and the full FCVGAN provides a clearer view of the contribution of the CVAE. On Kvasir-SEG, the Dice score increases from 0.8397 with FCM+GAN to 0.8590 with the full model. The largest difference is observed on ETIS-Larib, where the Dice score increases from 0.5412 to 0.7619 when the CVAE is included. On the other hand, FCM+GAN performs slightly better on CVC-ClinicDB (0.8976 vs. 0.8932) and Kvasir-Sessile (0.5082 vs. 0.4792). Therefore, the contribution of the CVAE is not uniform across all datasets, but it provides an important benefit for the overall performance and particularly for the generalization to ETIS-Larib.

Removing FCM clustering from the full architecture (CVAE-GAN) results in an average improvement of +11.28%. This configuration performs well on Kvasir-Sessile (+44.87%) and also improves Kvasir-SEG (+2.58%) and CVC-ClinicDB (+1.65%). However, it degrades the performance on ETIS-Larib by −4.00%. This suggests that the FCM component can contribute to the generalization of the model when transferring to a more challenging target distribution.

We also experiment with removing the GAN discriminator and keeping the CVAE configuration. In this case, the performance degrades substantially on ETIS-Larib, reaching −21.53%, while Kvasir-Sessile shows an improvement of +18.27%. The average improvement becomes −0.50%. This indicates that adversarial training plays an important role in generating images that are useful for downstream segmentation.

The GAN-only configuration achieves an average improvement of +7.51%. It improves CVC-ClinicDB by +2.36% and Kvasir-Sessile by +38.78%, but its performance decreases on ETIS-Larib by −11.36%. Therefore, although the GAN alone can provide useful augmentation on some datasets, its performance is less consistent across the different target domains.

Overall, the ablation results show that the contribution of the different components depends on the target dataset. The GAN discriminator plays an important role in maintaining useful image generation, while the FCM component contributes to the organization of the latent representation. The new FCM+GAN experiment also shows more clearly the contribution of the CVAE. Although removing the CVAE can still produce strong results on some datasets, especially Kvasir-Sessile, it considerably reduces the performance on ETIS-Larib and lowers the average improvement from +13.92% to +7.29%. These results suggest that the combination of FCM clustering, CVAE encoding, and adversarial training provides a more balanced augmentation framework across the evaluated datasets.

### 5.7. Comparison with Related Work

In this section, we compare our proposed approach (FCVGAN) with recent polyp synthesis methods for segmentation augmentation in Table 10. The comparison across methods is a bit challenging due to variations in segmentation backbones, training protocols, datasets, and evaluation metrics. Nevertheless, we organize the results by generative approach and highlight the main methodological differences. All improvement values (Δ) are computed from Dice scores. For FCVGAN, the reported Dice scores correspond to the mean performance across five independent training runs.

Among the compared methods, Polyp-DDPM [55] provides one of the most direct comparisons as it also employs UNet++ for segmentation evaluation. On Kvasir-SEG, FCVGAN achieves a mean Dice improvement of +2.4%, compared with +0.7% reported for Polyp-DDPM. Although the two methods use the same segmentation backbone, the training and evaluation protocols are not identical, and therefore this comparison should be interpreted with caution. Polyp-DDPM also reports performance degradation in some cross-dataset evaluations, while FCVGAN shows positive mean improvements across all four datasets evaluated in our transfer setting.

GAN-based approaches also show varying results. Adjei et al. [23] report a +3.0% Dice improvement on Kvasir-SEG using a modified Pix2Pix architecture with a U-Net backbone, which is comparable to the +2.4% mean improvement obtained by FCVGAN. However, their evaluation is limited to a single dataset without cross-dataset validation. MinimalGAN [42] reports improvements of +0.4% on CVC-ClinicDB and +2.2% on ETIS-Larib using PraNet as the segmentation backbone. FCVGAN achieves improvements of +0.8% and +6.2% on the same datasets, respectively. SemanticPolypGAN [52] also reports a +2.8% improvement on Kvasir-SEG using UACANet. These results show that GAN-based augmentation can provide useful improvements, although the magnitude of the benefit depends strongly on the dataset, segmentation backbone, and training protocol.

Among diffusion-based methods, ArSDM [54] achieves the highest absolute performance on CVC-ClinicDB with a Dice score of 0.933 and reports a substantial improvement of +20.2% on ETIS-Larib. However, its improvement on Kvasir-SEG is considerably smaller (+0.1%), which shows that the benefit of synthetic augmentation can vary substantially between datasets. ControlPolypNet [56] achieves an improvement of +2.95% on CVC-ClinicDB through ControlNet-based synthesis with a quality filtering mechanism, while its performance decreases by −0.66% on Kvasir-SEG. These results further show that even advanced generative approaches do not provide uniform improvements across all evaluation settings.

From this comparison, we gain several observations. First, methods using different segmentation backbones can achieve substantially different absolute Dice scores, which makes it difficult to separate the contribution of the generative model from the capability of the downstream segmentation network. Second, the effectiveness of synthetic augmentation is generally dataset-dependent, with several methods showing strong improvements on some datasets and limited or negative effects on others. Third, the experimental protocols differ considerably between studies, including the datasets, synthetic-to-real data ratios, segmentation architectures, and evaluation procedures, which limits direct ranking between the methods.

FCVGAN provides positive mean improvements across all four evaluated datasets under the transfer setting, with gains of +2.4%, +0.8%, +16.0%, and +6.2% on Kvasir-SEG, CVC-ClinicDB, Kvasir-Sessile, and ETIS-Larib, respectively. The largest relative improvement is observed on Kvasir-Sessile, which has the lowest baseline segmentation performance. Moreover, FCVGAN uses a single generator trained on Kvasir-SEG to augment several target datasets without additional generative model training. Overall, these results indicate that FCVGAN can provide useful synthetic augmentation across different target datasets, while the comparison with existing methods should be considered in light of the differences in backbones and experimental protocols.

## 6. Discussion and Conclusions

This study introduced FCVGAN, a generative architecture that combines Fuzzy C-Means clustering with a Conditional VAE-GAN for synthetic polyp image generation. Our evaluation across the four benchmark datasets provides several observations regarding the use of generative models for polyp segmentation augmentation.

The proposed architecture combines three main components. The SPADE-based generator enables control over the location, shape, and boundaries of the synthesized polyps by preserving the spatial information provided by the input segmentation masks. The multi-scale discriminator helps the generator reproduce realistic appearance and texture at different resolutions, while the FCM module organizes the latent representation through soft clustering. Together, these components allow FCVGAN to preserve the structure defined by the input masks while introducing variations in the generated images.

We evaluated two augmentation strategies: per-dataset training, where a separate generative model is trained for each dataset, and transfer learning, where a single FCVGAN model trained on Kvasir-SEG is used to generate synthetic images for all target datasets. Across five independent segmentation runs, transfer augmentation achieved the highest mean Dice score on three of the four datasets, with improvements of +2.4%, +0.8%, +16.0%, and +6.2% on Kvasir-SEG, CVC-ClinicDB, Kvasir-Sessile, and ETIS-Larib, respectively. Per-dataset augmentation performed best on Kvasir-Sessile, where an improvement of +20.3% was obtained. Although none of the pairwise comparisons remained statistically significant after correction for multiple comparisons, transfer augmentation showed large effect sizes compared with the baseline across all four datasets. Considering also that only five independent runs were available, these results are interpreted mainly based on the direction and magnitude of the effects rather than statistical significance alone.

An important observation is obtained when comparing generation quality with downstream segmentation performance. Per-dataset training achieved a better average FID score than transfer learning (135.48 compared with 173.43), indicating that its generated images were generally closer to the distribution of the corresponding real datasets. However, this did not consistently result in better segmentation performance, as transfer augmentation achieved the highest mean Dice score on three of the four datasets. The memorization analysis provides additional information about this behavior. Per-dataset generated images showed a higher average maximum correlation with the training data (0.926) compared with transfer-generated images (0.767), indicating stronger similarity to the training samples. These observations suggest that visual fidelity alone does not completely determine the usefulness of synthetic images for data augmentation and that diversity with respect to the original training data may also play an important role.

The robustness experiment further showed that the effect of synthetic augmentation depends on the type of image degradation. On ETIS-Larib, the transfer model achieved the highest Dice score on clean and blurred images, while the per-dataset model performed best under reduced brightness and contrast. Under Gaussian noise, however, the baseline model obtained the highest performance. Therefore, synthetic augmentation does not provide uniform robustness against all image-quality degradations, but it can improve performance under several acquisition-related variations.

The ablation study also showed that the contribution of the different FCVGAN components is dataset-dependent. The complete FCVGAN achieved an average improvement of +13.92% across the four datasets. Removing the CVAE branch while keeping FCM and adversarial training reduced the average improvement to +7.29%, with a particularly large degradation on ETIS-Larib. Removing FCM resulted in an average improvement of +11.28%, while the CVAE-only configuration produced an overall degradation of −0.50%. The GAN-only configuration achieved an average improvement of +7.51%. These results indicate that individual components can contribute differently depending on the target dataset. In particular, the complete FCVGAN provides the most balanced performance across the evaluated datasets rather than each component being uniformly beneficial in every case.

The comparison with related work further shows that the benefit of synthetic augmentation depends strongly on the generative method, segmentation backbone, dataset, and evaluation protocol. FCVGAN provides positive mean improvements across all four datasets in the transfer setting while using a single generator trained on Kvasir-SEG. However, direct ranking against existing methods should be interpreted carefully because the compared studies use different segmentation architectures and experimental settings.

From a sensing perspective, FCVGAN can be directly connected to the data-processing stage of endoscopic visual sensing. Colonoscopy images are acquired through endoscopic imaging systems, and the performance of automatic segmentation methods depends strongly on the quality and diversity of the acquired images. Variations in illumination, blur, noise, contrast, and other acquisition conditions can affect the visual information provided to the segmentation model. In this context, FCVGAN aims to complement the endoscopic sensing pipeline by generating additional mask-conditioned images that increase the diversity of the training data. The robustness experiments under different image degradations further show how the trained segmentation models behave when the quality of the acquired images is altered. Future work could extend this evaluation using images acquired from different endoscopic devices and acquisition settings to better study the generalization of the proposed approach across sensing systems.

## 7. Limitations and Future Work

Several limitations should be acknowledged. First, our evaluation relies only on publicly available benchmark datasets and uses a single segmentation backbone (UNet++). Therefore, the generalization of the observed improvements to other segmentation architectures and independent clinical datasets remains to be evaluated. In addition, patient- or video-level identifiers were not available in the dataset files used in our experiments, which prevented us from performing patient- or video-grouped resampling during the statistical analysis.

The transfer learning strategy also assumes the availability of a suitable source dataset. When the source and target domains differ substantially, the benefit of transfer augmentation may become more limited. Furthermore, the robustness analysis was performed using controlled image degradations rather than images acquired directly under different endoscopic devices or clinical acquisition conditions. Future work should therefore include external datasets collected from different endoscopic systems and imaging settings.

Another limitation concerns the memorization analysis. Maximum Pearson correlation was used as a practical indicator of image-level similarity, but it does not fully capture perceptual or feature-level similarity between generated and real images. Future work could extend this analysis using perceptual distances, deep feature representations, nearest-neighbor visualization, and region-specific similarity analysis.

FCVGAN also remains a relatively large generative model, mainly due to the SPADE-based generator. Although image generation is relatively fast during inference, future work could investigate lighter architectures, pruning, or knowledge distillation to reduce the computational requirements.

Finally, improved segmentation performance on benchmark datasets does not necessarily translate into improved clinical outcomes. This study did not evaluate adenoma detection rates, histological characterization, or endoscopist performance. Future work should include blinded evaluation of the generated images by multiple endoscopists, assessment of anatomical and pathological plausibility, and prospective validation on independent clinical data. Extending FCVGAN to other medical imaging modalities and incorporating domain adaptation strategies could also improve its applicability across different source and target domains.

## Figures and Tables

**Figure 1:**
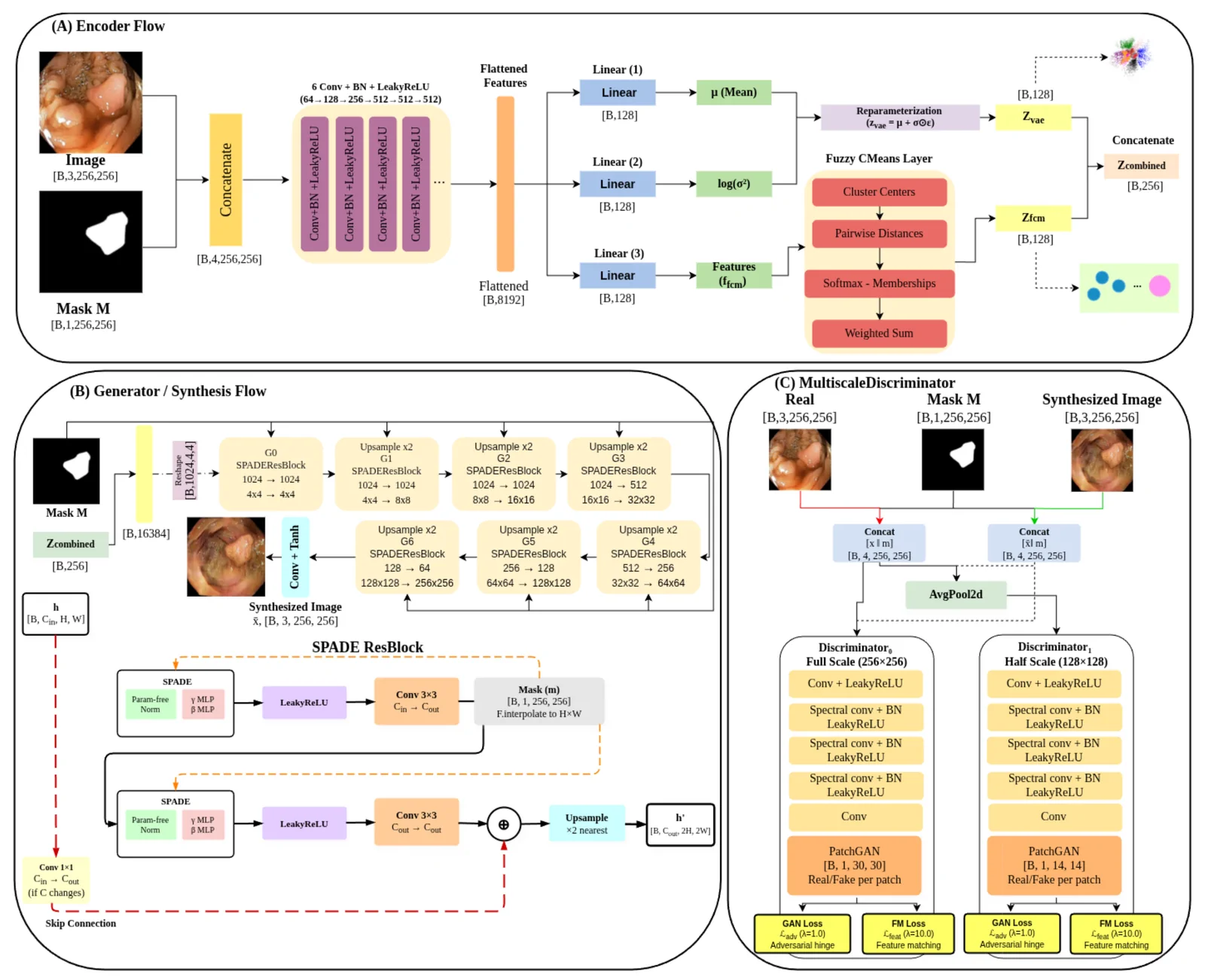
Overview of the proposed FCVGAN architecture. (**A**) The encoder extracts features from concatenated image–mask pairs and produces two latent components: $z_{\mathrm{vae}}$ through variational sampling and $z_{\mathrm{fcm}}$ through fuzzy clustering. (**B**) The generator progressively upsamples the combined latent code using SPADE residual blocks conditioned on the input mask. (**C**) The multi-scale discriminator evaluates realism at full and half resolutions using PatchGAN with feature matching.

**Figure 2:**
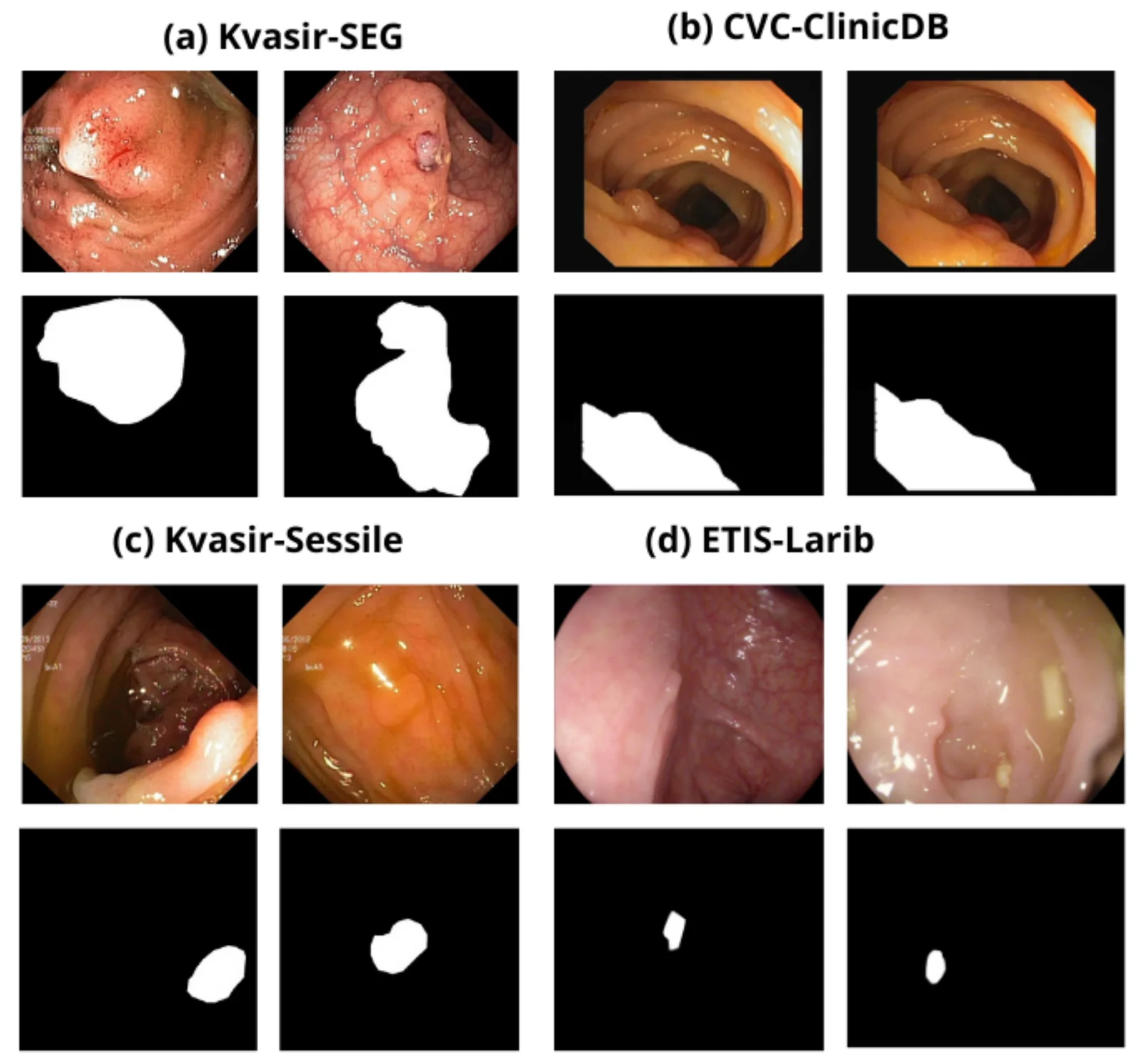
Representative samples from the four polyp segmentation datasets. Each panel shows two colonoscopy images (top) with corresponding ground truth masks (bottom). (**a**) Kvasir-SEG: large, prominent polyps with varied morphology. (**b**) CVC-ClinicDB: polyps captured under controlled imaging conditions. (**c**) Kvasir-Sessile: flat sessile polyps with subtle boundaries. (**d**) ETIS-Larib: small polyps with challenging imaging conditions including specular reflections.

**Figure 3:**
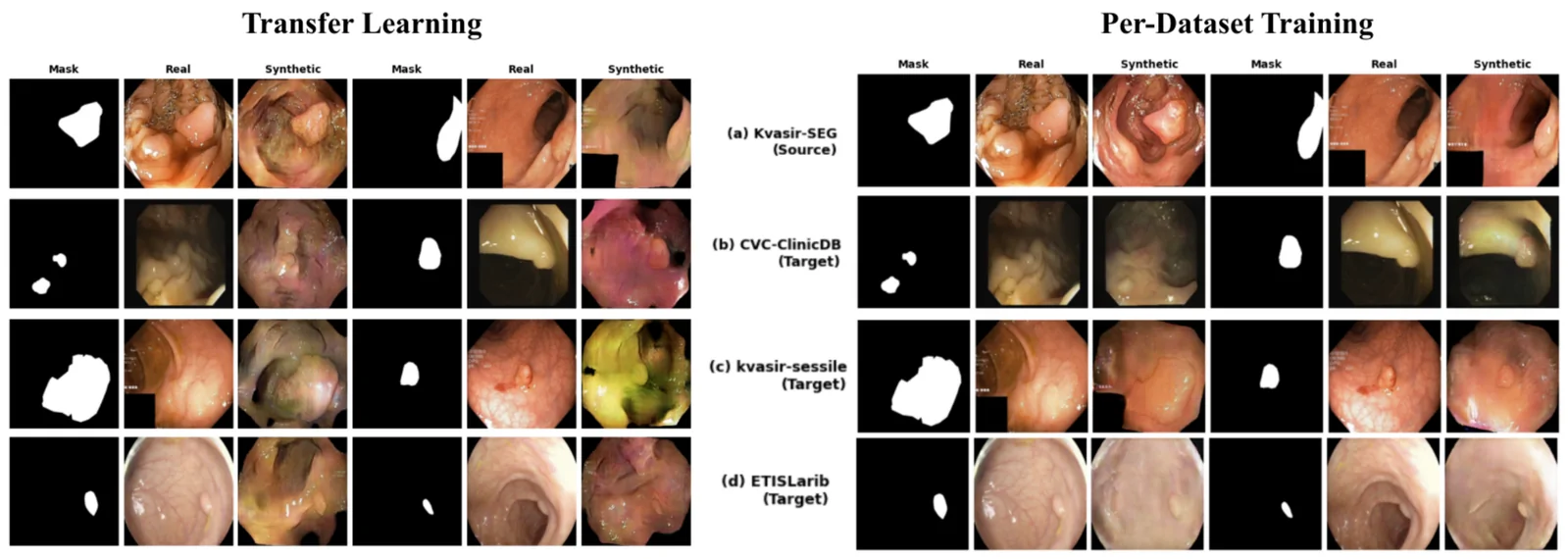
Qualitative comparison of synthetic polyp images generated by FCVGAN. **Left (transfer learning):** The model is trained on Kvasir-SEG (source) and applied to all datasets. **Right (per-dataset training):** Separate models are trained for each dataset. Rows: (**a**) Kvasir-SEG, (**b**) CVC-ClinicDB, (**c**) Kvasir-Sessile, and (**d**) ETIS-Larib. The per-dataset approach produces more visually consistent images, while transfer learning shows color artifacts on target datasets but generates more diverse samples.

**Figure 4:**
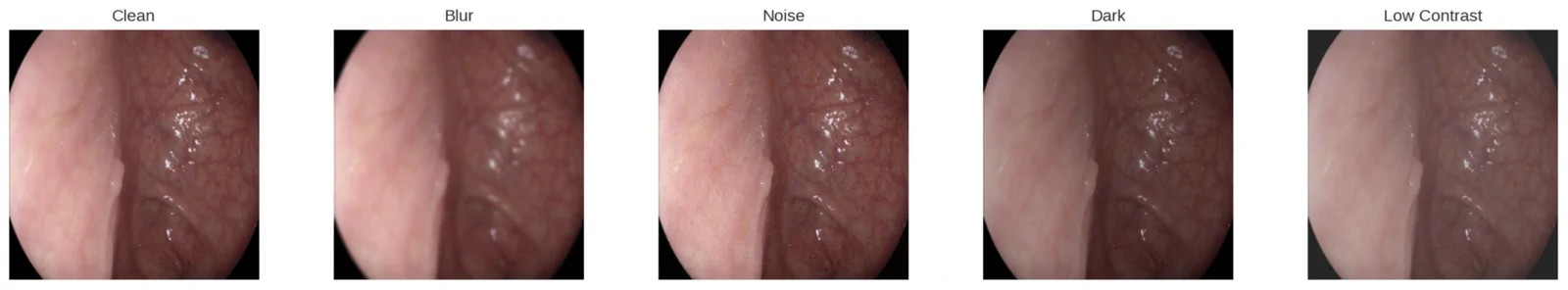
Examples of the image conditions used in the robustness evaluation on ETIS-Larib. From left to right: clean image, Gaussian blur, Gaussian noise, reduced brightness, and reduced contrast.

**Figure 5:**
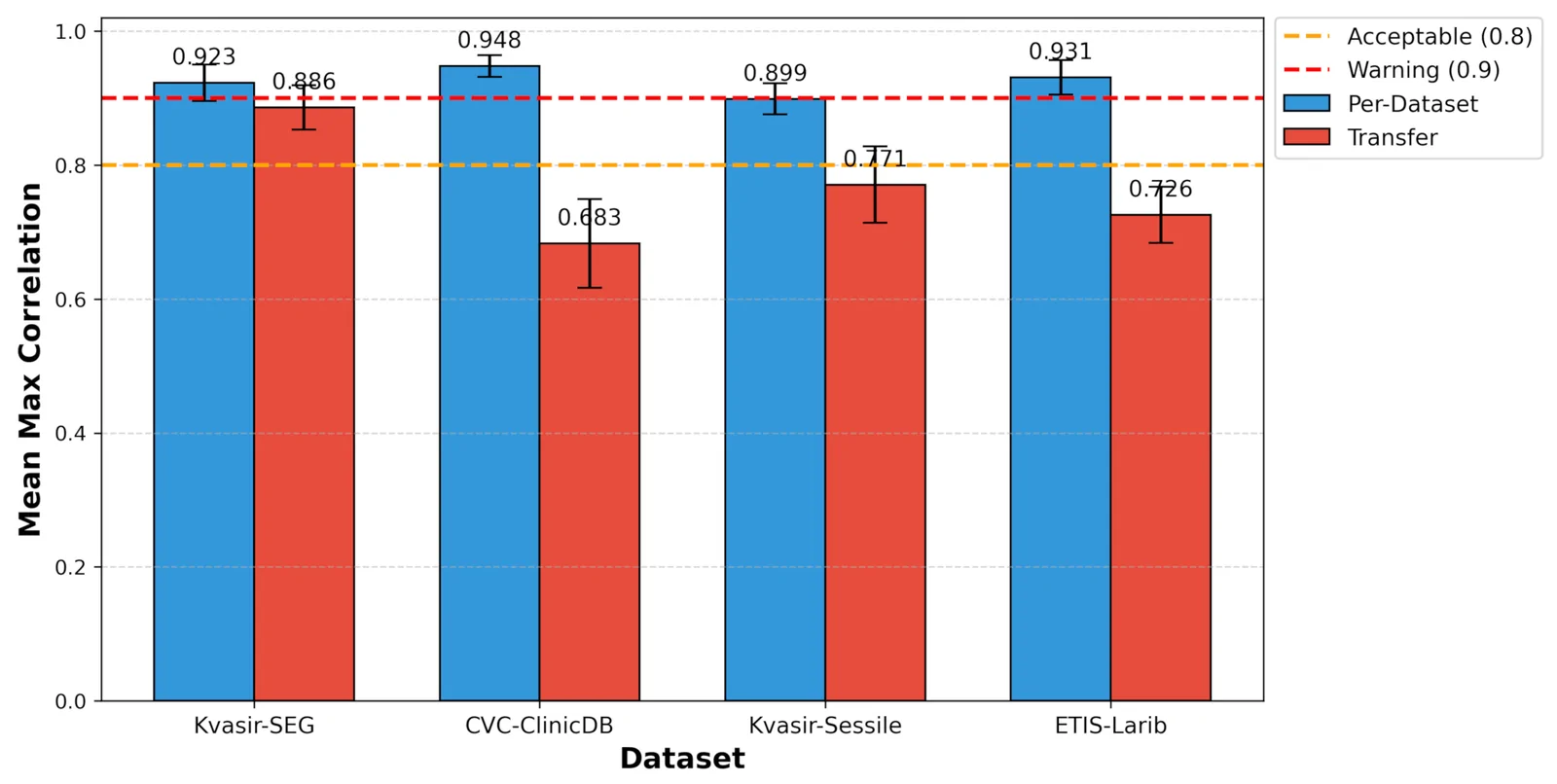
Memorization analysis comparing per-dataset and transfer learning approaches. Lower maximum correlation indicates greater diversity with respect to the training images.

**Table 1:** Summary of polyp segmentation datasets used in our experiments.

Dataset	Images	Train	Val	Test
Kvasir-SEG	1000	699	150	151
CVC-ClinicDB	612	428	91	93
Kvasir-Sessile	196	136	29	31
ETIS-Larib	196	135	30	31
Total	2004	1398	300	306

**Table 2:** Parameter distribution and active parameter counts of FCVGAN.

Component	Parameters	Description
*Encoder E*		
Convolutional layers	11.1 M	Feature extraction
FCM Module	1.05 M	Clustering layer + projection
VAE Latent	2.1 M	μ and $\log\sigma^{2}$ heads
**Encoder Total**	**14.25 M**	
Generator *G* (SPADE)	96.2 M	Spatially adaptive decoder
Discriminator *D*	14.0 M	Multi-scale PatchGAN
**FCVGAN Training Total**	**124.45 M**	E+G+D
**Per-Dataset Inference**	**110.45 M**	E+G
**Transfer Inference**	**96.2 M**	Generator only

**Table 3:** Computational cost (GFLOPs) per forward pass of FCVGAN components.

Component/Setting	Parameters	GFLOPs
Encoder *E*	14.25 M	2.02
Generator *G* (SPADE)	96.2 M	130.18
Discriminator *D*	14.0 M	3.25
Transfer inference (*G*)	96.2 M	130.18
Per-dataset inference (E+G)	110.45 M	132.20

**Table 4:** Comparison of baseline, transfer, and per-dataset FCVGAN augmentation strategies. Results are reported as mean ± standard deviation of the Dice coefficient across five independent training runs.

Dataset	Baseline	Transfer	Improv.	Per-Dataset	Improv.
Kvasir-SEG	0.8318 ± 0.0109	**0.8518 ± 0.0069**	+2.4%	0.8461 ± 0.0062	+1.7%
CVC-ClinicDB	0.8812 ± 0.0043	**0.8887 ± 0.0092**	+0.8%	0.8830 ± 0.0084	+0.2%
Kvasir-Sessile	0.4015 ± 0.0444	0.4659 ± 0.0560	+16.0%	**0.4832 ± 0.0662**	+20.3%
ETIS-Larib	0.7007 ± 0.0468	**0.7443 ± 0.0227**	+6.2%	0.7388 ± 0.0322	+5.4%

**Table 5:** Statistical comparison of baseline, transfer, and per-dataset augmentation across five independent training runs. ΔDice represents the mean paired difference between the second and first strategy in each comparison. CI denotes the 95% bootstrap confidence interval of the paired seed-level difference, *W* is the Wilcoxon signed-rank statistic, $p_{\mathrm{adj}}$ is the Bonferroni-adjusted *p*-value, and $r_{rb}$ is the matched-pairs rank-biserial correlation.

Dataset	Comparison	ΔDice	95% CI	*W*	*p*	$p_{\mathrm{adj}}$	$r_{\mathrm{rb}}$ (Effect)
Kvasir-SEG	Baseline vs. Transfer	+0.0200	[0.0105, 0.0285]	0	0.0625	0.750	+1.000 (Large)
	Baseline vs. Per-dataset	+0.0143	[0.0037, 0.0281]	1	0.1250	1.000	+0.867 (Large)
	Transfer vs. Per-dataset	−0.0058	[−0.0157, 0.0036]	4	0.4375	1.000	−0.467 (Medium)
CVC-ClinicDB	Baseline vs. Transfer	+0.0075	[0.0006, 0.0143]	2	0.1875	1.000	+0.733 (Large)
	Baseline vs. Per-dataset	+0.0018	[−0.0044, 0.0101]	7	1.0000	1.000	−0.067 (Negligible)
	Transfer vs. Per-dataset	−0.0056	[−0.0181, 0.0071]	4	0.4375	1.000	−0.467 (Medium)
Kvasir-Sessile	Baseline vs. Transfer	+0.0644	[0.0160, 0.1151]	1	0.1250	1.000	+0.867 (Large)
	Baseline vs. Per-dataset	+0.0816	[0.0217, 0.1655]	1	0.1250	1.000	+0.867 (Large)
	Transfer vs Per-dataset	+0.0173	[−0.0733, 0.0822]	5	0.6250	1.000	+0.333 (Medium)
ETIS-Larib	Baseline vs Transfer	+0.0437	[−0.0006, 0.0846]	2	0.1875	1.000	+0.733 (Large)
	Baseline vs. Per-dataset	+0.0382	[0.0021, 0.0790]	2	0.1875	1.000	+0.733 (Large)
	Transfer vs. Per-dataset	−0.0055	[−0.0310, 0.0157]	7	1.0000	1.000	+0.067 (Negligible)

**Table 6:** Robustness evaluation on ETIS-Larib under different image degradation conditions. Dice values are reported as mean ± standard deviation across five independent training runs. Δ represents the performance drop relative to the clean condition.

Condition	Baseline		Transfer		Per-Dataset	
	Dice	Δ	Dice	Δ	Dice	Δ
Clean	0.7007±0.0468	–	0.7443±0.0227	–	0.7388±0.0322	–
Gaussian blur	0.4206±0.0823	0.2800±0.0536	0.4957±0.0524	0.2486±0.0417	0.4845±0.0418	0.2543±0.0455
Gaussian noise	0.2113±0.0338	0.4894±0.0760	0.1899±0.0146	0.5545±0.0359	0.1739±0.0352	0.5649±0.0530
Reduced brightness	0.0947±0.0393	0.6060±0.0343	0.2008±0.0512	0.5436±0.0532	0.2482±0.0259	0.4906±0.0243
Reduced contrast	0.0567±0.0594	0.6439±0.0326	0.2578±0.0623	0.4866±0.0712	0.2851±0.0357	0.4538±0.0424

**Table 7:** Generation quality metrics comparing per-dataset and transfer learning approaches. Lower FID indicates better quality. Best results per dataset in **bold**.

Dataset	Approach	FID ↓	SSIM ↑	PSNR ↑
Kvasir-SEG	Per-Dataset	**68.91**	0.354	11.32
Kvasir-SEG	Transfer	126.58	**0.367**	**11.89**
CVC-ClinicDB	Per-Dataset	**107.27**	**0.545**	**14.49**
CVC-ClinicDB	Transfer	189.37	0.289	12.14
Kvasir-Sessile	Per-Dataset	**170.62**	**0.387**	**12.06**
Kvasir-Sessile	Transfer	186.74	0.331	11.85
ETIS-Larib	Per-Dataset	195.11	**0.466**	**11.68**
ETIS-Larib	Transfer	**191.02**	0.353	11.25
**Average**	Per-Dataset	**135.48**	**0.438**	**12.39**
**Average**	Transfer	173.43	0.335	11.78

*Note:* ↓ indicates that lower values are better, while ↑ indicates that higher values are better.

**Table 8:** Memorization analysis based on the maximum Pearson correlation between each synthetic image and the real training images. Lower values indicate more diverse generation.

Dataset	Approach	Mean ± Std	Max	>0.9
Kvasir-SEG	Per-Dataset	0.923 ± 0.027	0.970	84.4%
	Transfer	**0.886 ± 0.033**	0.946	35.9%
CVC-ClinicDB	Per-Dataset	0.948 ± 0.016	0.980	99.1%
	Transfer	**0.683 ± 0.066**	0.810	0.0%
Kvasir-Sessile	Per-Dataset	0.899 ± 0.023	0.935	49.3%
	Transfer	**0.771 ± 0.057**	0.865	0.0%
ETIS-Larib	Per-Dataset	0.931 ± 0.026	0.970	86.7%
	Transfer	**0.726 ± 0.042**	0.824	0.0%
**Average**	Per-Dataset	0.926	0.964	79.9%
	Transfer	**0.767**	0.861	9.0%

**Table 9:** Ablation study comparing segmentation improvement (%) across different model configurations using transfer learning. Results correspond to a single training run for each configuration.

Dataset	FCVGAN (Full)	FCM + GAN	CVAE-GAN	CVAE	GAN Only
Kvasir-SEG	+2.98	+0.67	+2.58	+0.30	+0.26
CVC-ClinicDB	+1.24	+1.75	+1.65	+0.98	+2.36
Kvasir-Sessile	+37.43	+45.74	+44.87	+18.27	+38.78
ETIS-Larib	+14.02	−19.01	−4.00	−21.53	−11.36
**Average**	**+13.92**	+7.29	+11.28	−0.50	+7.51

**Table 10:** Comparison with recent polyp synthesis methods for segmentation augmentation. Improvements (Δ) are relative to the corresponding baseline using real data only and are computed from Dice scores. FCVGAN results are reported as mean ± standard deviation across five independent runs. Methods vary in segmentation backbones and evaluation protocols, limiting direct comparison. Only Polyp-DDPM uses the same backbone (UNet++) as our method.

Method	Type	Backbone	Dataset	Dice	IoU	Δ
**FCVGAN (Ours)**	VAE-GAN	UNet++	Kvasir-SEG	0.8518±0.0069	–	+2.4%
			CVC-ClinicDB	0.8887±0.0092	–	+0.8%
			Kvasir-Sessile	0.4659±0.0560	–	+16.0%
			ETIS-Larib	0.7443±0.0227	–	+6.2%
*GAN-based Methods*						
Adjei et al. [23]	Pix2Pix	U-Net	Kvasir-SEG	0.850	0.776	+3.0%
MinimalGAN [42]	Style-GAN	PraNet	CVC-ClinicDB	0.926	0.873	+0.4%
			ETIS-Larib	0.649	0.575	+2.2%
SemanticPolypGAN [52]	Style-GAN	UACANet	Kvasir-SEG	0.916	0.857	+2.8%
*Diffusion-based Methods*						
Polyp-DDPM [55]	DDPM	UNet++	Kvasir-SEG	0.834	0.716	+0.7%
ArSDM [54]	Diffusion	PraNet	CVC-ClinicDB	0.933	0.885	+3.8%
			ETIS-Larib	0.755	0.681	+20.2%
			Kvasir-SEG	0.899	0.845	+0.1%
ControlPolypNet [56]	ControlNet	TransNetR	CVC-ClinicDB	0.820	0.749	+2.95%
			Kvasir-SEG	0.858	0.786	−0.66%

## Data Availability

The datasets used in this study are publicly available from the original sources cited in the manuscript. The synthetic data generated during this study have not been deposited in a public repository. Further inquiries can be directed to the corresponding author.

## Abbreviations

The following abbreviations are used in this manuscript:

CVAE	Conditional Variational Autoencoder
FCM	Fuzzy C-Means
FCVGAN	Fuzzy C-Means Conditional Variational Autoencoder GAN
GAN	Generative Adversarial Network
IoU	Intersection over Union
KL	Kullback–Leibler
SPADE	Spatially Adaptive Denormalization
VAE	Variational Autoencoder
